# Deep learning as a highly efficient tool for digital signal processing design

**DOI:** 10.1038/s41377-024-01599-8

**Published:** 2024-09-11

**Authors:** Andrey Pryamikov

**Affiliations:** Prokhorov General Physics Institute of the Russian Academy of Sciences, Moscow, Russia

**Keywords:** Optics and photonics, Optical techniques

## Abstract

The backpropagation algorithm, the most widely used algorithm for training artificial neural networks, can be effectively applied to the development of digital signal processing schemes in the optical fiber transmission systems. Digital signal processing as a deep learning framework can lead to a new highly efficient paradigm for cost-effective digital signal processing designes with low complexity.

It is known that historically long-haul optical communication systems have operated at the limits of electronic technology. Also, the performance of long-haul optical communications systems is degraded by such impairments as chromatic dispersion, polarization mode dispersion (PMD) and Kerr fiber nonlinearities. However, over the past two decades, due to advances in silicon technology, there have appeared data converters^1^ whose operating speed is commensuarate with current optical line rates permitting the use of digital signal processing (DSP). The powerful compensation and effective mitigation of the transmission impairments can be obtained by using DSP for high-speed fiber-optic communication systems. The symbiotic combination of DSP, spectrally efficient modulation formats and coherent detection has resulted in digital coherent optical receivers. Such digital coherent optical receivers offer improved sensitivity and allow to overcome optical linear impairments of high-speed systems (chromatic dispersion and PMD^2,3^).

Although DSP in coherent receivers can effectively compensate for linear transmission impairments, compensation of nonlinear effects remains a separate big problem and is a limiting factor for high capacity signals^4^. Fiber nonlinearity mitigation techniques can be applied at the coherent receivers and since the advent of DSP the great progress has been made with DSP-based nonlinearity mitigation. It is worth noting that the DSP-based nonlinearity mitigation is more resilient against link conditions. It is also important that the improvement of nonlinear characterisitcs can be assessed against the initial level, at which the linear effects have been effectively minimized. Failure to comply with this requirement may lead to distorted performance assesments depending on linear compensation criteria.

DSP operates block-by-block with static and dynamic equalizers designed to counteract chromatic dispersion and PMD in the fiber link. However, these equalizers may demonstrate limited performance given the comprehensive and global nature of the fiber optics communications effect, due to the lack of global cooperation. Since traditional DSP is based on 2 samples/symbol processing then the complexity reduction of existing DSP architectures at low processing rate while maintaining exceptional performance remains a very challenging task. One way to solve these problems is to use deep learning (DL) models which have the advantage of a data-driven approach^5,6^ which allows to process signals without the need for expert knowledge.

Deep learning (DL) or artificial intelligence is an extensive scientific discipline which enables computer sytems to solve problems via imitating complex biological processes (such as learning, reasoning and self-correction). For example, since the optical fibers exhibite nonlinear behavior, the channels in the optical fiber communication sytems interfere in a nonlinear way both with other channels on the same fiber and interact among themselves. In this case, DL can be applied to estimate the strength of nonlinear interaction and mitigate its effects. Also, DL can help with modeling and optical network control. The key technology of DP models is gradient descent through the backpropagation algorithm. The main problem here is that the DSP framework is incompatible with DL techniques due to its reliance on model-driven solutions. In some works^7^, the DP and DSP blocks are considered as separate entities and the optimization process is mainly related to the DL algorithms. At the same time, redesigning the entire DSP platform to include DL techniques for performance improvement may not be effective in terms of costs. Therefore it would be attractive to achieve improved performance using DL techniques which don’t require significant changes to the existing DSP platform.

In a newly published paper in Light: Science & Applications^8^, Zekun Niu, Hang Yang, Lyu Li, Minghui Shi, Suozhi Xu, Weisheng Hu and Lilin Yi from the State Key Lab of Advanced Optical Communication Systems and Networks, School of Electronic Information and Electrical Engineering, Shanghai Jiao Tong University, China, have proposed a DSP scheme with a DL optimization framework called learnable DSP (LDSP) (Fig. 1). The LDSP architecture implementation makes for easier communication between DSP modules that use the backpropagation algorithm, providing a comprehensive compensation of global signal impairments and thereby increasing DSP efficiency. All LDSP blocks jointly process information allowing full use of DSP resources for linear compensation. Technically, LDSP is a traditional DSP framework with online training that uses a combination of expertise and DL benefits. Besides, a high symbol rate processing can be accomplished with a minimal bit error rate. Thus, LDSP not only increases performance, but also demonstrates a significant 48% reduction in complexity compared to a 2 samples/symbol processing in regular systems. Сompared to traditional DSP optimized by hyperpaprameters, a noticeable increase in quality factor of ~1.21 dB for a 400 Gbit/s signal after transmission over 1600 km of fiber transmission was experimentally demonstrated using a combination of LDSP and the nonlinear disturbance compensation algorithm.

**Fig. 1:**
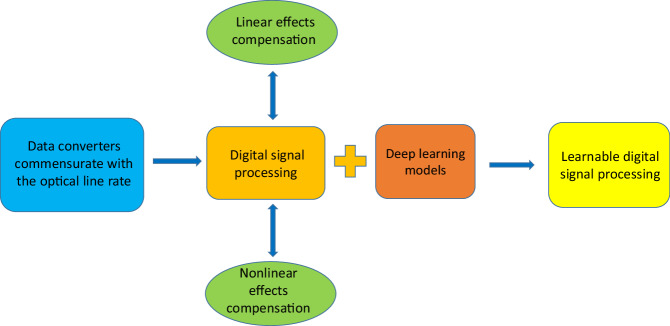
Schematic view of DSP evolution in optical communication systems and Learnable Digital Signal Processing

The authors believe that the methods discussed in this study are relevant to the broader field of DSP, which is attracting significant attention in various fiber optic communication scenarios, including short-reach, medium-reach, and long-haul links.
